# Prevalence of Metabolic Syndrome in Patients with Psoriasis

**DOI:** 10.1100/2012/312463

**Published:** 2012-04-24

**Authors:** Ilkin Zindancı, Ozlem Albayrak, Mukaddes Kavala, Emek Kocaturk, Burce Can, Sibel Sudogan, Melek Koç

**Affiliations:** Department of Dermatology, Goztepe Training and Research Hospital, Istanbul, Turkey

## Abstract

*Background*. Psoriasis is a chronic inflammatory skin disorder in which proinflammatory cytokines including IL-6 and TNF-**α** increase both locally and systematically. It is thought that chronic inflammation results in metabolic diseases and proinflammatory cytokines give rise to the development of atherogenesis, peripheral insulin resistance, hypertension, and type 2 diabetes. Our aim was to investigate the prevalence of metabolic syndrome in patients with psoriasis vulgaris. *Methods*. Study consisted of 115 plaque-type psoriasis patients and 140 healthy individuals. Data including body weight, height, waist circumference, body-mass index, and arterial blood pressure were collected. Fasting blood glucose, triglyceride, and HDL levels were determined. International Diabetes Federation Criteria for Metabolic Syndrome and Insulin Resistance were used for evaluating patients with metabolic syndrome and diabetes. *Results*. Compared to the control group, metabolic syndrome, diabetes mellitus, and hypertension were found to be higher in psoriasis patients. Metabolic syndrome was increased by 3-folds in psoriasis patients and was more prevalent in women than in men. It was determined that the prevalence of metabolic syndrome was higher in psoriasis patients after the age of 40. Metabolic syndrome was not related to smoking, severity of psoriasis, and duration of disease. *Conclusions*. Our findings suggest that psoriasis preconditions occurrence of a group of diseases such as diabetes mellitus, hypertension, and metabolic syndrome. For this reason, patients with psoriasis should be treated early and they should be followed with respect to metabolic diseases.

## 1. Introduction

Psoriasis is a chronic inflammatory skin disorder which is characterized by local and systemic escalation of proinflammatory cytokines such as IL-6 and TNF-*α* [1, 2]. Proinflammatory cytokines which take place in chronic inflammation are accused of augmentation of atherogenesis and peripheric insulin resistance and thus leading to hypertension and type II Diabetes Mellitus (DM) [2, 3]. Recent research reported an association between psoriasis and metabolic disorders such as obesity, dyslipidemia, and type II DM and it is shown that severe psoriasis might be associated with increased mortality rate due to cardiovascular disorders [2, 4–6]. Since psoriasis is characterized by a systemic inflammation which involves numerous cytokines and inflammatory markers in particular TNF-*α*, it may predispose the patients to metabolic disorders. We aimed to investigate the prevalence of metabolic syndrome in patients with psoriasis vulgaris.

## 2. Patients and Methods

The study included 115 psoriasis vulgaris patients and 140 healthy, sex, and age-matched control subjects who do not have psoriasis and any systemic disease. Inclusion criteria for cases were age more than 18 years, clinical diagnosis of chronic plaque psoriasis (lasting at least 6 months), and not receiving any systemic or local antipsoriatic treatment for the last 4 weeks. Informed consent was given by all the patients who enrolled in the study. Information sheets for the patients included age, gender, weight, height, body mass index (BMI), waist circumference, smoking habits, blood pressure, age of onset, and duration of the disease. Exclusion criteria were pregnancy and psoriatic arthritis. Severity of psoriasis was assessed by psoriasis area severity index (PASI) and accepted as severe in patients with PASI > 10 and mild/moderate in patients with PASI ≤ 10 [7]. BMI was calculated as weight/height (kg/m^2^) and metabolic syndrome was diagnosed in the presence of central obesity in addition to two or more criteria of the International Diabetes Foundation: waist circumference ≥94 cm in men or ≥80 cm in women; hypertriglyceridemia ≥150 mg/dL; HDL <40 mg/dL in men or <50 mg/dL in women; blood pressure ≥130/85 mmHg; fasting blood glucose ≥100 mg/dL [8]. Triglyceride and HDL levels were calculated by enzymatic assay, glucose was calculated by glucose oxidase assay. Student's *t*-test, Mann-Whitney *U*, chi-square, and Fisher's exact chi-square tests were used for statistical analysis of the data. Enter logistical regression analysis was used for multivariate determination of parameters affecting metabolic syndrome. *P* values <0.05 were accepted as statistically significant and confidence interval (CI) was 95%.

## 3. Results

The study group included 64 (55.7%) females and 51 (44.3%) males with a mean age of 45.4 (range 19–79) years. The control group consisted of 70 (50%) females and 70 (50%) males with a mean age of 43.4 (range 19–70) years. Duration of the disease was 11.4 ± 9.2 years, and mean age of onset were 34.24 ± 17.3 years. Severity of psoriasis was mild to moderate in 76 (66.1%) of the patients and severe in 39 (33.9%) of the patients with a mean PASI of 9.98 ± 8.6. There was a significant difference between the smoking rates of the groups which were 35.7% and 24.3% in psoriasis and control groups, respectively (*P* < 0.05). Waist circumference ≥94 cm in men and ≥80 cm in women was observed in 73% and 83.6% of psoriasis and control subjects, respectively; waist circumference was significantly slimmer in psoriasis patients (*P* < 0.05). Mean BMI was 28.73 ± 6.12 kg/m^2^ and 28.22 ± 4, 9 kg/m^2^ in psoriasis and control groups, respectively. No significant differences were found between groups with respect to BMI (*P* > 0.05). There were also no significant differences between groups with respect to high triglyceride levels and low HDL levels (*P* > 0.05). In contrast, we found a significant correlation between psoriasis and hypertension, diabetes mellitus and metabolic syndrome. Hypertension and diabetes mellitus were significantly more common in psoriasis patients as compared to controls. We found a higher prevalence of MS in psoriasis patients than in controls (53% versus 39%) (*P* < 0.01). The prevalence of various components of MS in cases and controls along with *P* values is given in Table 1.

Although all patients with MS had waist circumference ≥80 cm in females and ≥94 cm in males, there was no significant difference with respect to mean BMI between psoriasis patients and control cases with MS (*P* > 0.05). But we observed that female psoriasis patients with MS had a higher BMI (30.60 ± 7.18 in females versus 26.37 ± 3.37 in males) (*P* < 0.01).

Considering the data for diabetes mellitus, hypertension, and MS in psoriasis patients, it appeared that deviations from normal were most prevalent at the fifth decade of life (Table 2). No apparent differences were noted regarding the prevalence of DM and hypertension between the genders (*P* > 0.05). In contrast, we found a significant correlation between MS and female gender. MS was more common in female patients than male patients (*P* < 0.05). The mean age of psoriasis onset in patients with MS was significantly higher than patients without MS (*P* < 0.01). Prevalence of MS was not correlated to smoking, severity of psoriasis, and duration of disease (*P* > 0.05) (Table 3).

Multiple logistic regression analysis revealed that MS is increased by 2.94-fold in psoriasis patients (95% CI : 1.40–6.19). When the parameters that might influence MS in psoriasis patients such as age, gender, age of onset, and smoking were evaluated, the model was found highly significant (*P* < 0.001) with a Negelkerke (*R* ^2^) value of 0.293 and determination coefficient of 72.2%. The influence of female gender and age on the occurrence of MS in psoriasis patients was found significant (*P* < 0.01). Female gender increased the risk of MS by 3.195-fold (95% CI : 1.12–9.04) compared to males; patients aged between 40–49 had an increased risk of MS by 5.141-fold (95% CI : 1.75–24.47), patients aged over 60 had an increased risk of MS by 6.531-fold (95% CI : 1.55–3.37). It was found that MS was independent from smoking habit (Table 4).

## 4. Discussion

This study reveals that hypertension, diabetes mellitus, and MS are significantly more common in psoriasis patients than controls. Therefore, all dermatologists should be aware of MS or individual components of MS might be associated with psoriasis during the course of this disease. Recent studies showed that psoriasis is associated with metabolic disorders such as hypertension, type II DM, dyslipidemia, abdominal obesity, insulin resistance, and cardiac disorders and the risk of metabolic syndrome is increased in patients with psoriasis [2–6, 9–12]. Sommer et al. reported that there is a significant association between psoriasis and type II DM, hypertension, hyperlipidemia, and coronary artery disease and MS is increased by twofolds in a study they conducted in 581 patients [3]. Other studies showed an increased frequency of ischemic heart disease, DM, hypertension, and dyslipidemia in patients with psoriasis when compared to controls [13, 14]. Gisondi et al. found increased prevalence of hypertrygliceridemia and MS in psoriasis patients compared to controls, but they did not find any difference between psoriasis patients and controls with respect to low levels of HDL, DM, and hypertension [15]. Farshchian et al. failed to demonstrate any difference between psoriasis patients and controls with regard to fasting blood glucose, triglyceride, cholesterol, HDL, LDL, and VLDL levels [16]. In our study we observed that psoriasis is associated with smoking, DM, hypertension, and MS. DM and hypertension was accompanying our psoriasis patients along with MS. These findings confirmed the literature [3, 11, 13, 14]. It is reported that smoking is more prevalent in psoriasis patients [13–15]. We found the rates of smoking higher than controls. Levels of triglyceride and cholesterol were reported to be high in psoriasis patients [3, 13, 14]. We did not detect any dyslipidemia in our patients. This was consistent with previous findings which found normal lipid levels in psoriasis patients and concluded that hyperlipidemia did not have a clinical significance in psoriasis patients [15, 16]. It has been reported that hypertension and DM were common in psoriasis patients regardless of gender and hypertension had an increased frequency by advanced age while DM might be seen in any age [3, 13, 14]. In our study, we found both hypertension and DM in advanced age, regardless of gender.

Although obesity is reported more frequent among psoriasis patients, we have not found BMI and waist circumference significantly higher in psoriasis patients [3, 13, 14]. However we observed a higher prevalence of MS among psoriasis patients than controls. This may be explained by the literature which concluded that the association between psoriasis and MS is independent from the tendency of psoriatic patients to be obese [15].

In our study we observed that MS was increased by 3-fold in psoriasis patients. The association of psoriasis with MS was independent from smoking. These findings confirmed those in the literature [11, 13, 14, 17]. We found the prevalence of MS increased by 5-fold in patients aged between 40 and 59 years. This was consistent with previous studies by Sommer et al. and Gisondi et al. who reported that MS was significantly higher in psoriasis patients after the age of 40 [3, 15]. Cohen et al. also demonstrated that the association between psoriasis and MS was pronounced after the age of 50 [13]. However Nisa and Qazi observed the higher prevalence of MS in psoriasis patients in the age group 18–30 [17].

In contrast to the reports that MS was regardless of gender, we observed a significant relationship between MS and gender [3, 11, 15, 17]. MS was more prevalent in women and female gender increased the prevalence of MS by 3-fold.

High BMI levels were frequent in our female patients with MS and this finding was confirming the previous findings [3]. We believe that high frequency of MS in female patients is the result of increased waist circumference due to high BMI.

It was reported that MS is related to the duration of the disease, psoriasis starts in young ages in patients with MS and duration of the disease is longer in patients with MS [3, 15]. But we observed that psoriasis started at advanced age in our patients with MS and MS was not related to the duration of the disease. There are controversies in the literature about the association of MS with severity of the disease. Sommer et al. reported a positive relation with severity, while Gisondi et al. and Nisa and Qazi declared an independent relation [3, 15, 17]. Like Gisondi et al. and Nisa and Qazi, we did not observe an association between severity of the disease and MS.

Our results showed that psoriasis predisposes to the development of DM, hypertension, and MS and psoriasis increased the prevalence of MS by 3-fold. Therefore, we recommend evaluating psoriasis patients for the presence of metabolic diseases which may interfere with the patients' health for the rest of their living.

## Figures and Tables

**Table 1:** Metabolic syndrome criteria in psoriasis patients and controls.

		Psoriasis (*n* = 115)	Control (*n* = 140)	*^++^* *P*
		*n* (%)	*n* (%)	
Waist circumference (cm)	M ≥ 94; F ≥ 80	84 (73.0%)	117 (83.6%)	0,041*
	M < 94; F < 80	31 (27.0%)	23 (16.4%)	

BMI (kg/m^2^)		28.73 ± 6.1	28.22 ± 4.9	0.473

Triglyceride (mg/dL)	≥150	52 (45.2%)	55 (39.3%)	0.340
	<150	63 (54.8%)	85 (60.7%)	

HDL (mg/dL)	F ≤ 50; M ≤ 40	50 (43.5%)	46 (32.9%)	0.082
	F > 50; M > 50	65 (56.5%)	94 (67.1%)	

BP (mmHg)	≥130/85	47 (40.9%)	33 (23.6%)	0.003**
	<130/85	68 (59.1%)	107 (76.4%)	

FBG (mg/dL)	≥100	56 (48.7%)	49 (35.0%)	0.027*
	<100	59 (51.3%)	91 (65.0%)	

MS	(+)	61 (53.0%)	59 (39.0%)	0.004**
	(−)	54 (47.0%)	91 (65.0%)	

^++^Chi-square test, *P < 0.05, **P < 0.01.

BMI: Body Mass Index; MS: Metabolic syndrome; F: Female; M: Male; BP: Blood pressure; FBG: Fasting blood glucose.

**Table 2:** Association of MS, DM, and HT with age in psoriasis patients.

		*n*	Age			*P*
			Min-max	Med	SD	
MS	(+)	59	18–79	51.28	14.43	0.001**
	(−)	56	17–79	39.16	14.05	

DM	(+)	56	23–79	52.62	14.13	0.001**
	(−)	59	17–77	38.50	13.44	

HT	(+)	47	27–79	54.76	13.07	0.001**
	(−)	68	17–71	38.89	15.55	

*^+^*Student's* t*-test, **P < 0.01.

**Table 3:** The associations of MS with patient characteristics in psoriasis patients.

		MS		
Psoriasis group		(+)	(−)	*P*
		M ± SD (Median)	M ± SD (Median)	
^+^Duration of disease		12.29 ± 9.65	10.42 ± 8.55	0.277
^+^Age of onset		39.73 ± 17.03	28.03 ± 15.56	0.001**

		*n* (%)	*n* (%)	^++^ *P*

Gender	Male	21 (41.2%)	24 (58.8%)	0.023*
	Female	40 (62.5%)	30 (37.5%)	

	No	28 (54.9%)	23 (45.1%)	
	Ex-smoker	12 (66.7%)	6 (33.3%)	0.166
Smoking	Active smoker	17 (41.4%)	24 (58.6%)	
	Passive smoker	4 (80.0%)	1 (20.0%)	

PASI	Mild-moderate	39 (51.3%)	37 (48.7%)	0.604
	Severe	22 (56.4%)	17 (43.6%)	

*^+^*Student's* t*-test*, ^ ++^*Chi-square test, *P < 0.05, **P < 0.01.

**Table 4:** Multiple regression analysis.

				95,0% C.I. for Exp (*B*)		
	*B*	S.E.	Sig.	Exp (*B*)	Lower	Upper
Gender: F	1.161	0.531	0.029*	3.195	1.12	9.04
Age			0.007**			
Age: 40–59	1.637	0.549	0.003**	5.141	1.754	15.06
Age: ≥60	1.877	0.733	0.010*	6.531	1.552	24.47
Age of onset ≥ 40	0.095	0.572	0.869	1.099	0.358	3.376
Smoking			0.417			
Ex-smoker	0.894	0.731	0.221	2.446	0.583	10.25
Active smoker	0.056	0.533	0.917	1.057	0.372	3.007
Passive smoker	1.275	1.236	0.241	0.476	0.138	1.644
